# Prediction of septic and hypovolemic shock in intensive care unit patients using machine learning

**DOI:** 10.5935/0103-507X.20220280-en

**Published:** 2022

**Authors:** Stela Mares Brasileiro Pessoa, Bianca Silva de Sousa Oliveira, Wendy Gomes dos Santos, Augusto Novais Macedo Oliveira, Marianne Silveira Camargo, Douglas Leandro Aparecido Barbosa de Matos, Miquéias Martins Lima Silva, Carolina Cintra de Queiroz Medeiros, Cláudia Soares de Sousa Coelho, José de Souza Andrade Neto, Sóstenes Mistro

**Affiliations:** 1 Postgraduate Program in Collective Health, Universidade Federal da Bahia - Vitória da Conquista (BA), Brazil.; 2 Postgraduate Program in Pharmaceutical Assistance, Universidade Federal da Bahia - Salvador (BA), Brazil.; 3 Universidade Federal da Bahia - Vitória da Conquista (BA), Brazil.; 4 Postgraduate Program in Medicine and Health, Universidade Federal da Bahia - Salvador (BA), Brazil.; 5 Faculdades Santo Agostinho - Vitória da Conquista (BA), Brazil.; 6 Department of Pharmacy, Complexo Hospitalar de Vitória da Conquista - Vitória da Conquista (BA), Brazil.; 7 Universidade Estadual do Sudoeste da Bahia - Vitória da Conquista (BA), Brazil.

**Keywords:** Machine learning, Shock, septic, Shock, Algorithms, Decision tree, Inpatients, Critical care, Intensive care units

## Abstract

**Objective:**

To create and validate a model for predicting septic or hypovolemic shock from easily obtainable variables collected from patients at admission to an intensive care unit.

**Methods:**

A predictive modeling study with concurrent cohort data was conducted in a hospital in the interior of northeastern Brazil. Patients aged 18 years or older who were not using vasoactive drugs on the day of admission and were hospitalized from November 2020 to July 2021 were included. The Decision Tree, Random Forest, AdaBoost, Gradient Boosting and XGBoost classification algorithms were tested for use in building the model. The validation method used was k-fold cross validation. The evaluation metrics used were recall, precision and area under the Receiver Operating Characteristic curve.

**Results:**

A total of 720 patients were used to create and validate the model. The models showed high predictive capacity with areas under the Receiver Operating Characteristic curve of 0.979; 0.999; 0.980; 0.998 and 1.00 for the Decision Tree, Random Forest, AdaBoost, Gradient Boosting and XGBoost algorithms, respectively.

**Conclusion:**

The predictive model created and validated showed a high ability to predict septic and hypovolemic shock from the time of admission of patients to the intensive care unit.

## INTRODUCTION

The evolution of a patient to shock is one of the main concerns of health teams in intensive care units (ICUs), as it represents one of the most frequent causes of death in these units.^(1)^ Early identification of the condition and prompt initiation of treatment have been the most effective measures to reduce mortality associated with shock. However, the work dynamics in ICUs, especially when there is a high occupancy rate and a large number of critically ill patients, can be a barrier to the identification of signs of shock within the ideal time window. This difficulty, often observed in routine ICUs, has been a stimuli for the expressive growth of tools that can optimize time and resources to obtain better clinical results in patients in intensive care.^(2)^

Septic shock may affect up to 35% of patients admitted to the ICU, and mortality in these cases reaches 63%.^(3)^ In addition to the high death rate, the occurrence of septic shock is associated with the development of physical and cognitive sequelae, resulting from a long stay in the ICU, as well as a reduction in the quality of life with constant hospitalizations and a significant increase in health costs.^(4,5)^ Hypovolemic shock, in turn, despite having lower overall mortality, is also a cause of death, especially in the ICUs of trauma hospitals, with a mortality rate approaching 19%.^(6)^

The infusion of intravenous fluids and the rapid initiation of antimicrobial therapy are considered effective in reducing the risk of evolution to shock in high-risk patients.^(7,8)^ In individuals with sepsis and hypotension, for example, the infusion of fluids results in improved perfusion and increased mean arterial pressure (MAP),^(9)^ which may reduce the chance of progression to in-hospital death by up to 2.7% for each 1% extra fluid administered, provided that the identification of the condition and the initiation of treatment occur in a timely manner.^(10)^ Likewise, the immediate initiation of antibiotics in cases of sepsis significantly reduces the risk of death, as mortality rates increase by 10% for every hour of delay in starting treatment.^(11)^ These data demonstrate the need for identification and prioritization in the surveillance of patients with high potential for evolution to shock in ICUs, which can be strongly supported by a tool that is easy to apply and with high accuracy.

The predictive models created from machine learning algorithms are used as a basis for the creation of tools with increasing application in the health care field.^(12)^ They are used to predict several clinically relevant conditions, including sepsis and septic shock.^(13)^ However, the models used to predict shock use a large number of variables, which are generally difficult to obtain and may be difficult to reproduce in other scenarios.^(13)^ Some models use variables that are more easily collected but are exclusive to the prediction of septic shock and with predictor variables collected after patient admission.^(14)^ These characteristics make the application of these models in the daily clinical practice of ICUs limited due to the lack of practicality and unavailability of data when necessary.

Thus, this study aimed to create and validate a model for predicting septic or hypovolemic shock with easily obtainable variables collected at admission from patients admitted to the ICU.

## METHODS

This was a predictive modeling study conducted with data from patients admitted to the ICU of a hospital located in the northeast region of Brazil. At the time of the study, the unit had 20 beds and received patients with various clinical and surgical conditions. All patients aged 18 years or older who were not using vasoactive drugs (VAD) on the day of admission and were admitted to the ICU between November 2020 and July 2021 were included in the study. Patients are incomplete data for any of the variables used in the study were excluded from the analyses. Data collection from the medical records was performed daily, from admission to discharge of the patient from the ICU, with the aid of a questionnaire prepared by the research team on the KoBoToolbox platform^(15)^ through the KoCoCollect Android app. The collected data were audited daily to avoid loss or error in their collection or entry.

### Target variable

The occurrence of septic or hypovolemic shock was assessed using VAD, norepinephrine and/or vasopressin at some point during hospitalization in the ICU. Although the definition of septic shock is the use of VAD to maintain MAP greater than 65 mmHg and serum lactate greater than 2mmol/L,^(16)^ for the present study, shock was assessed only by the use of VAD during hospitalization. This strategy was used due to the absence of lactate levels for most patients. Although there is the possibility of overestimating the number of patients with shock, the need for VAD is an alert condition that can be avoided as long as it is signaled in a timely manner for the implementation of measures that can prevent shock. The target variable consisted of a dichotomous variable, with yes or no values for the use of VAD during hospitalization, except for admission.

### Predictors

The mining step of the predictor variables was restricted to the data available on the date of admission of the patient to the ICU, which resulted in the identification of 12 variables: “age”, “presence of infection”, “use of orotracheal tube”, “use of urinary catheter”, “use of central venous catheter”, “use of catheter for invasive blood pressure monitoring”, “sedation”, “Simplified Acute Physiology Score III (SAPS)”, “temperature”, “systolic blood pressure”, “comorbidities” and “heart rate”. The variables “temperature”, “systolic blood pressure” and “heart rate” were used as continuous variables, while the variable “SAPS” was categorized into ≤ 57 and > 57, according to a Brazilian study, in which the cutoff ratio of 57 showed better sensitivity and specificity in predicting hospital mortality.^(17)^ Patients with missing data were excluded from the analyses. We chose not to perform data imputation because only a small number of patients had missing data, with no impact on the predictive capacity of the model.

### Model training and validation

For the construction of the model, the data were imported into the Jupyter Notebook software, and the Pandas, Scikit-Learn and Matplotlib libraries of the Python language were used to create the model. The Decision Tree, Random Forest, AdaBoost, Gradient Boosting and XGBoost algorithms were tested in the search for the best result in the prediction of septic and hypovolemic shock. Other algorithms, such as artificial neural networks and logistic regression, were tested and presented inferior results with precision and recall less than 60%, while tree-based algorithms presented results greater than 80% in the evaluation metrics.

For model validation, the *k-fold cross validation* method was used. In this method, the database was subdivided into five datasets. In each of the five validations, a different part of the model was randomly chosen to represent the test group, and the rest of the data formed part of the training set. The final evaluation metrics are the arithmetic means of the five results obtained at the end of each validation. Although the dataset is not unbalanced, we chose not to use accuracy; therefore, the metrics of recall, precision and area under the curve were used (AUC) Receiver Operating Characteristic (ROC) for model evaluation. These metrics were chosen with the aim of reducing the number of false-positives, given the severity of the condition to be detected, as well as false negatives, to reduce the possibility of inadequate allocation of resources in the ICU.

This study was approved by the Research Ethics Committee of the *Universidade Federal da Bahia*, Multidisciplinary Institute of Health - *Campus Anísio Teixeira*, under number 38332720.4.0000.5556. The application of the Free and Informed Consent Form (ICF) was waived as all information was collected from the medical records and with minimal risk to patients.

## RESULTS

A total of 731 patients met the study inclusion criteria, of whom 11 were excluded because they had missing data on one or more predictor variables, which resulted in the inclusion of 720 patients for the analyses. The demographic data and general characteristics of the study population are described in table 1.

**Table 1:** General characteristics of the study population

Variables	
Age	67 [24]
Sex	
Female	277 (38,5)
Male	443 (61,5)
Comorbidities	
Yes	376 (52,2)
No	344 (47,8)
Location before ICU	
Emergency	308 (42,8)
Surgical center	146 (20,3)
Infirmary	143 (19,9)
Shock room	79 (11,0)
Another hospital	44 (6,0)
Length of stay	5 [7]

ICU - intensive care unit. The results are expressed as the median [interquartile range] or n (%).

**Figure 1:**
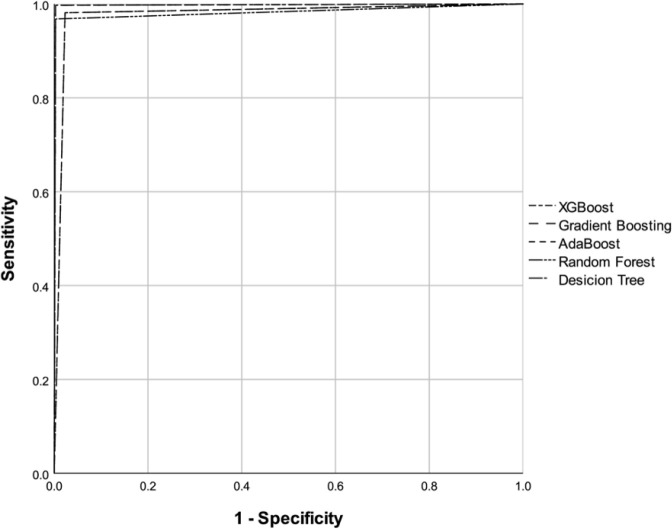
Receiver Operating Characteristic curve of the models in the prediction of shock

**Figure 2:**
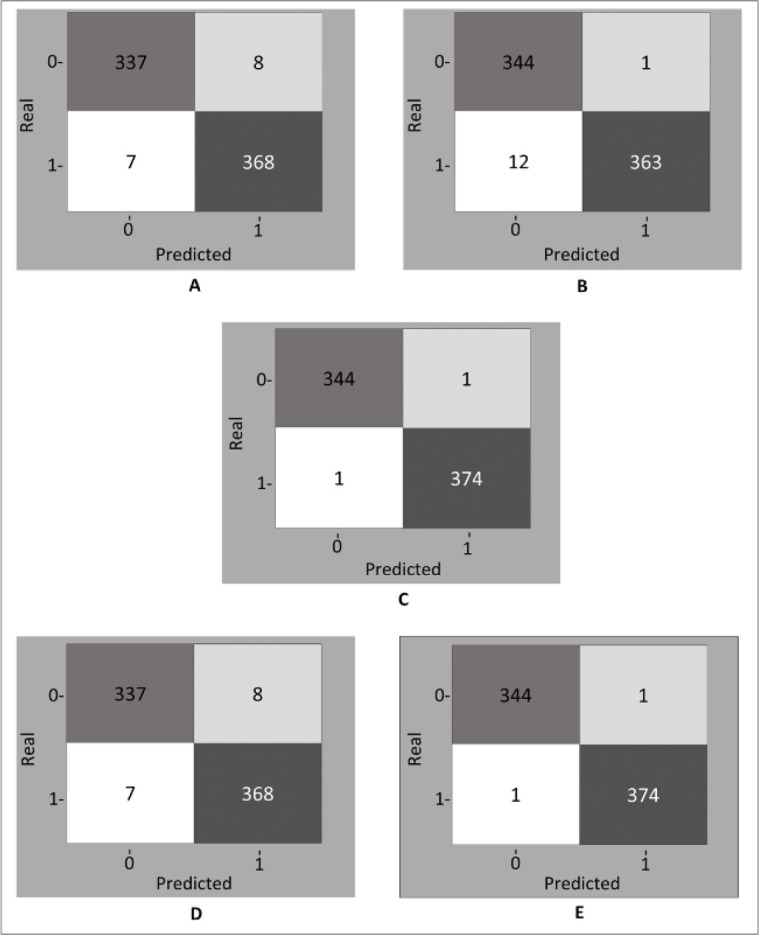
Confusion matrix of each model evaluated in the prediction of shock: (A) Decision Tree, (B) Random Forest, (C) XGBoost, (D) AdaBoost, and (E) Gradient Boosting.

Among the models compared, the best *recall* and thus the lowest number of false negatives were observed with the *Gradient Boosting* and *XGBoost* algorithms. The evaluation metrics of the models are described in table 2. The ASC-ROC and the confusion matrix of each model are presented in figures 1 and 2, respectively.

**Table 2:** Metrics for evaluating the performance of the models in predicting shock

Algorithm	Recall	Precision
Decision Tree	0.98	0.97
Random Forest	0.98	0.96
AdaBoost	0.97	0.97
Gradient Boosting	0.99	0.99
XGBoost	0.99	0.99

**Figure 3:**
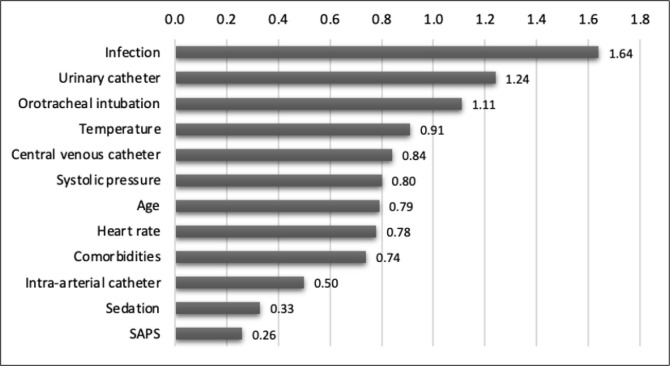
Importance of the variables for the predictive model

The model with the *XGBoost* algorithm presented better performance when considering the three evaluation metrics, with an ASC-ROC of 1.00. The importance of the variables for the predictive model was measured by calculating the mean and standard deviation of the decrease in impurity in each generated tree using the attribute feature importance. The variables that most contributed to the prediction in this algorithm were infection, urinary catheter, orotracheal intubation and temperature; the importance of the variables for the model is described in figure 3.

SAPS - Simplified Acute Physiology Score.

## DISCUSSION

Based on easy-to-obtain data, we developed and validated a prediction model that was able to correctly classify 99% of patients who would progress to septic or hypovolemic shock at some point during their stay in the ICU.

The direct correlation between the time of onset of symptoms, introduction of therapeutic actions and mortality associated with shock is a concept widely disseminated among intensive care teams. However, in the routine of an ICU, it is not uncommon for there to be a delay in the identification of the initial signs of shock and in the triggering of the set of measures that can reduce the chance of evolution to death. This delay may be related to both work overload and failures in the planning and systematization of care. Consequently, despite knowing when and how to act, the ideal time for diagnosis can be missed. Among the main potentials of our shock prediction model is its possibility of application as a support tool in the organization of the care process in the ICU, such as in defining the number and interval of nursing visits to the bed of a patient, based on his or her risk of evolution to shock, as well as the expansion of infectious surveillance, with monitoring of temperature, WBC and C-reactive protein, in addition to constant review of antimicrobial therapy, with escalation, when necessary.

Fluid administration is one of the main interventions for increasing tissue perfusion and reducing the progression to shock. This measure is used not only to avoid septic shock but also hypovolemic shock. Although the definition of the best fluid to be used remains under discussion, there is already sufficient evidence demonstrating that the earlier the infusion, the better the patient outcomes. Intravenous fluid therapy in patients who have sepsis without shock was responsible for the increase in MAP and was associated with shorter mechanical ventilation and ICU stay.^(18)^ Thus, the identification of a patient at risk of shock appears to be essential for the initiation of fluid resuscitation, which results in improved patient outcomes and decreases the risk of late hypotension. However, such conduct should be performed with caution so that there is no water overload or harm to critically ill patients.^(19)^ Therefore, the model that we validated in the present study may be valuable input for the decision-making of the care team about vigorous hydration of a patient in the ICU, even before the onset of the initial signs of hypotension or shock.

Some models have been proposed for the prediction of sepsis and septic shock in the ICU. However, these models have reproducibility limitations, mainly due to their dependence on a large set of variables. Some models include up to 20 different variables, some of which are impractical to obtain in the usual routine of an ICU, such as fibrinogen levels.^(20)^ Thus, the development of a prediction model composed of a reduced number of easily obtainable variables allows its reproduction in other hospitals, including those with limited resources.

A predictive model for the use of VAD, which also uses easily obtainable variables, was recently developed. However, its prediction concerns the use of vasopressors within 24 hours after ICU admission to aid in the initial management of these patients, as the predictors were vital signs that are usually available before ICU admission.^(21)^ Although a significant percentage of patients require VAD at the beginning of hospitalization, those who experience shock after the first 48 hours remain in the ICU for a longer period of time.^(22)^ Therefore, our model has the widest application potential and the possibility of contributing to the reduction of ICU length of stay.

A recent discussion regarding the use of *machine learning* algorithms to create predictive models in the health care field is about the interpretability of these tools.^(23)^ A model is considered interpretable when its decision-making process is easily explainable.^(24)^ The best model for predicting septic and hypovolemic shock in the present study was achieved using the XGBoost algorithm, a poorly interpretable algorithm. However, the variables used in our model are known to be associated with shock, and therefore, although the model is difficult to understand, the included variables make the model easily understandable for the end user in the decision-making process. This characteristic expands the possibility of practical application of the model validated in this study.

Our study has some limitations. One of them was the use of VAD alone to define shock due to the lack of serum lactate values; therefore, the number of patients with shock may be overestimated. However, the use of VAD already characterizes a scenario with the need for greater monitoring and care. Another limitation of our study is the number of patients included in the model. However, because it was a prospectively fed database with constant auditing of the data, there was a minimal loss of information, which resulted in variables with a high degree of completeness and, consequently, a reduction in the potential for bias produced by the size of the population studied. Likewise, the values obtained in the evaluation metrics suggest that the number of patients did not affect the performance of the model. In addition to the number of patients included, because this was a single-center study, it is not possible to say that our model can be applied to other ICUs. Therefore, it will be necessary to test its accuracy in different scenarios until it can be applied as a tool to support decision-making.

## CONCLUSION

The creation and validation of a predictive model based on an XGBoost classification algorithm showed high accuracy in predicting septic and hypovolemic shock from the moment of admission of patients to the intensive care unit based on variables that can be easily collected. This tool has the potential for application in the daily practice of intensive care teams as support for the organization of the care process to reduce the chance of evolution to shock in patients admitted to the intensive care unit. In addition, the model can be easily used to develop an application that can be accessed by professionals during their work routines.
